# Enantioselective Preparation of (*N*,*O*)-, (*N*,*N*)- and (*N*,*S*)-Spiroketal Moieties

**DOI:** 10.3390/molecules30204100

**Published:** 2025-10-15

**Authors:** Mata Sow, Edouard Fauran, Laurent Commeiras

**Affiliations:** Aix Marseille Univ, CNRS, Centrale Med, ISM2, Marseille, France

**Keywords:** spirocyclic, spiroketal, spiroaminal, enantioselective synthesis

## Abstract

This review highlights enantioselective strategies for preparing (*N*,*O*)- (*N*,*N*)- and (*N*,*S*)-spiroketal skeleton, which are key structural units found in many natural products and pharmacologically active substances. The strategies are mainly based on metallo-, organo- or Lewis acid-catalyzed cycloaddition or annulation reactions.

## 1. Introduction

**Figure 1:**
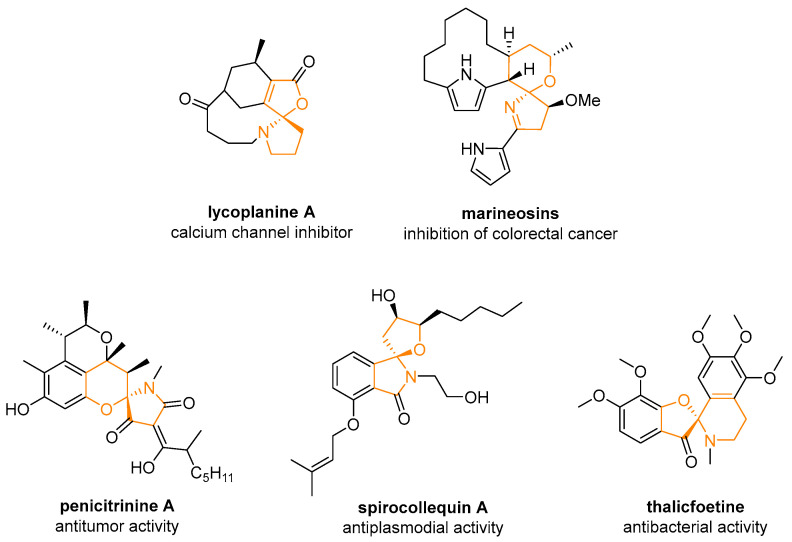
Selected natural products with spiroaminal skeleton.

According to the International Union of Pure and Applied Chemistry (IUPAC), spirocyclic compounds are defined as “compounds having one atom (usually a quaternary carbon) as the only common member of two rings” [1]. These molecules have attracted considerable interest due to the unique properties imparted by the spirocyclic quaternary carbon stereocenter, which confers conformational rigidity to the structure [2,3,4,5,6,7,8]. Since nitrogen-containing heterocyclic scaffolds represent a crucial class of compounds, it is not surprising that a wide variety of aza-spiroheterocyclic molecules are found in biologically active compounds and natural products. Among them, (*N*,*O*)-spiroketal derivatives are no exception (Figure 1). Compared to racemic strategies for the construction of such spiroaminals [9], the enantioselective construction of (*N*,*O*)-spirocenters is challenging due to its instability and propensity for racemization, even in mildly acidic conditions. In this review, we would like to describe progress in the field of enantioselective synthesis of such derivatives, as well as less studied (*N*,*N*)- and (*N*,*S*)-spiroketals.

## 2. (*N*,*O*)-Spiroketals

(*N*,*O*)-Spiroketal scaffolds represent a distinct class of spiroheterocyclic frameworks found in both natural and synthetic bioactive molecules. Their uniquely rigid three-dimensional architecture, along with their capacity to influence the physicochemical and pharmacokinetic properties of drug candidates, has driven increasing interest among synthetic and medicinal chemists. This has motivated the development of enantioselective strategies, including metallo-, Lewis acid- and organocatalyzed activation, for constructing these challenging spiroheterocyclic moieties.

### 2.1. Metallocatalyzed [4 + 2] Cycloaddition Reaction

**Scheme 1:**
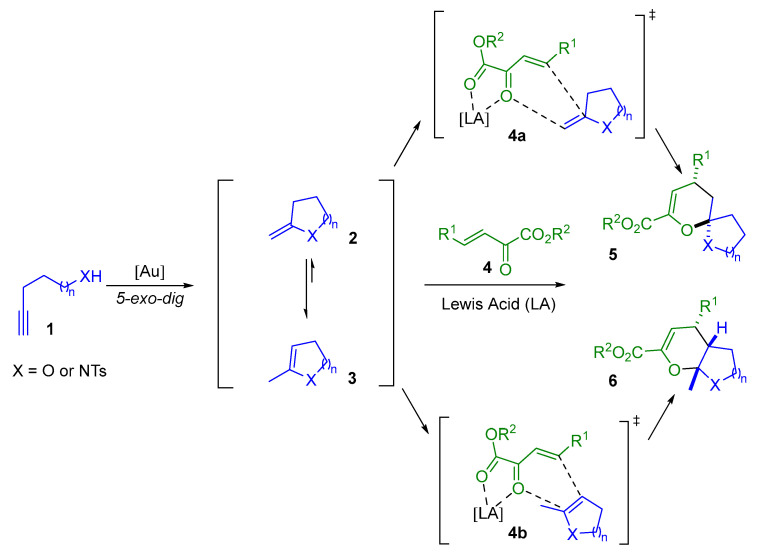
Racemic inverse electron-demand hetero-Diels–Alder.

Alkynyl alcohols and amides **1** are versatile precursors that can undergo 5-*exo*-dig cyclization in the presence of a gold(I) catalyst to generate enol-ether or enamine-ether intermediates **2**. These electron-rich alkene intermediates can be engaged in situ in an inverse electron-demand hetero-Diels–Alder reaction (IED hetero-DA reaction) with Lewis acid-activated α,β-unsaturated keto esters **4**, leading to the formation of (*O*,*O*)- or (*N*,*O*)-spiroketals **5**. A key challenge in this reaction is the control of the isomerization of the intermediate **2** to form the more thermodynamically stable species ***3*** (Scheme 1). This process can lead to the formation of fused (*O*,*O*)- or (*N*,*O*)-ketals **6** instead of the spirocyclic products **5** [10,11,12,13].

**Scheme 2:**
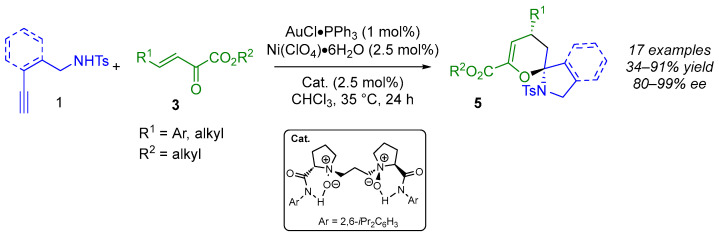
Enantioselective synthesis of spiroaminals via gold/nickel sequential catalysis.

In 2016, Liu and Feng demonstrated that introducing a bulkier chiral ligand to the Lewis acid favored the reactivity of the less sterically hindered enamine-ether ***2*** over ***3*** in the IED hetero-DA reaction. This shifted the isomerization equilibrium towards ***2***, thereby enhancing the formation of the desired spirocyclic products. Therefore, using an enantioselective relay catalytic system based on the combination of achiral π-acid gold(I) catalysis and chiral Lewis acid *N*,*N’*-dioxyde nickel(II) catalysis (Scheme 2), the authors obtained the desired spiroaminal ***5*** in good yields (34–91%), with high diastereoselectivity and excellent enantioselectivity (96–99%) [14]. It is worth noting that alkynyl amides were found to be more reactive than alkynyl alcohols, and that the spiroaminal products were obtained with higher yields and greater diastereo- and enantioselectivities. The lowest enantioselectivity (80%) was observed with phenyl-substituted alkynyl amide **1**.

It is worth noting that Kang reported a gold(I)/rhodium(III) bimetallic relay catalysis to furnish the spiroaminal using a similar strategy. Unlike spiroketals, which were obtained with excellent enantioselectivity, this approach was less effective for spiroaminal moieties [15].

### 2.2. Metallocatalyzed (4 + 2) Annulation Reaction

**Scheme 3:**
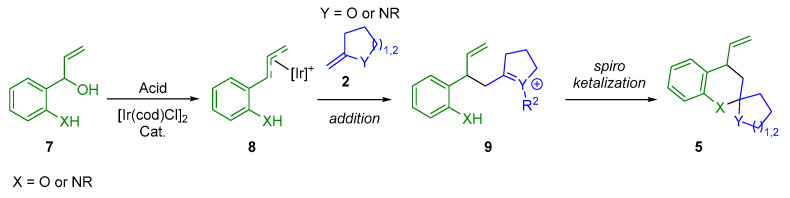
General route for iridium-catalyzed synthesis of (*N*,*O*)-spiroketals.

In the previous example (Scheme 2), an IED hetero-DA reaction involving vinyl enamides **2** and α,β-unsaturated keto esters **3** was used to form spiroaminal moieties **5**. Another method of accessing these motifs involves a cascade reaction comprising an addition reaction followed by a spiroacetali*z*ation reaction (Scheme 3) [16,17,18,19]. This methodology involves a reaction between a nucleophile **2** (an exocyclic vinyl enol ether or an exocyclic vinyl enamine) and an amphiphilic compound **8** that has both an electrophilic and a nucleophilic site. These amphiphilic species **8**, which are chiral π-allyl-Ir intermediates, can be obtained via iridium-mediated dehydration of 2-(1-hydroxyallyl)phenols or 2-(1-hydroxylallyl)anilines **7** in the presence of an acid promoter.

**Scheme 4:**
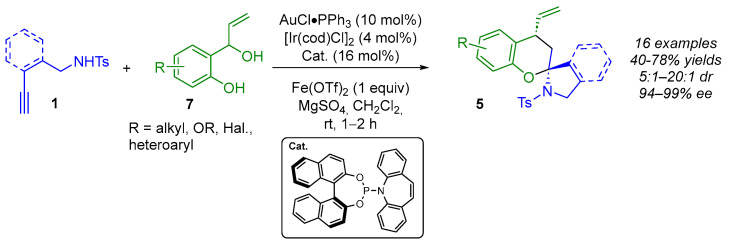
Enantioselective synthesis of spiroaminals via gold/iridium sequential catalysis.

In 2022, Deng and Yang investigated the use of amphiphilic reagents alongside alkynyl amides to create (*N*,*O*)-spiroketals **5** (Scheme 4) [16]. In the presence of in situ-generated gold(I)-cataly*z*ed vinyl enamides **2** from **1**, the desired spiroaminal products **5** were obtained via an enantioselective addition/spiroacetali*z*ation process. Under optimized conditions, **5** were obtained with good yields (40–78%) and high diastereo- and enantioselectivities (5:1–20:1 dr, 94–99% ee). The lowest diastereoselectivity (5:1–10:1) was observed when the aromatic ring of **7** was substituted in position **3**.

**Scheme 5:**
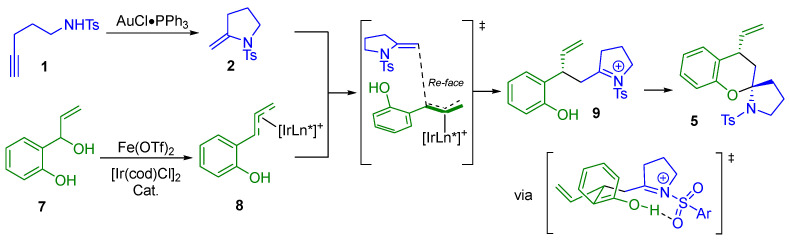
Proposed mechanism for the enantioselective synthesis of spiroaminals via gold/iridium sequential catalysis.

From a mechanistic point of view (Scheme 5), the first step is the gold(I)-catalyzed 5-*exo*-dig intramolecular hydroamination, which produces the corresponding exocyclic enamide **2**. Simultaneously, in the presence of Fe(OTf)_2_, the chiral Ir(I) complex would produce chiral π-allyl-Ir intermediates **8**, which would react with the exocyclic enamine to give the allylation intermediate **9**. Due to the shielded *Si*-face of **7**, enamide **2** would approach **7** from the *Re*-face. Finally, the spirocyclization step would occur via a chair-like Zimmerman–Traxler transition state, giving the desired (*N*,*O*)-spiroketal **5**.

**Scheme 6:**
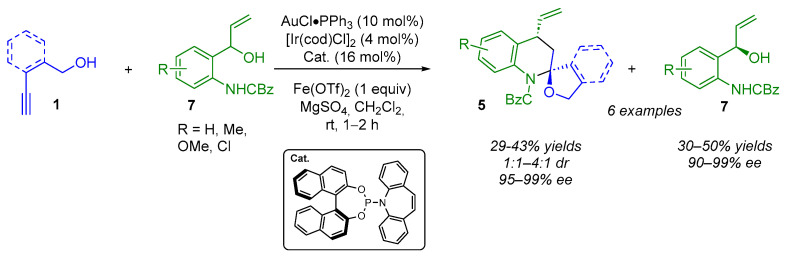
Enantioselective synthesis of spiroaminals from 2-(1-hydroxylallyl) anilines.

Interestingly, when using 2-(1-hydroxylallyl)anilines **7** as pre-amphiphilic species and alkynyl alcohols **1** (Scheme 6), the authors observed a kinetic resolution of **7** leading to both spiroaminal moieties **5** (with average diastereoselectivities of 2:1–4:1 and excellent enantioselectivities of 95–99% ee) and the enantiomerically enriched (90–99% ee) residual alcohols **7**.

**Scheme 7:**
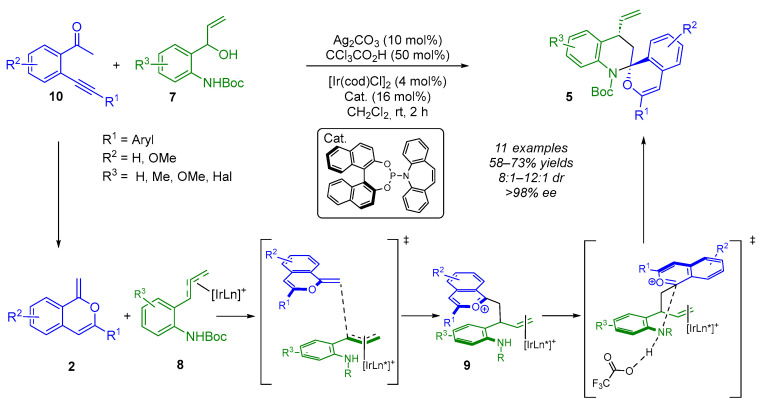
Enantioselective synthesis of spiroaminals from 2-(1-hydroxylallyl)anilines and methyleneisochromenes.

The same group extended this methodology for use with electron-rich methyleneisochromenes **2** as exocyclic enol ethers (Scheme 7). These are formed in situ from ortho-alkynylacetophenones **10** via a 6-*endo*-dig cycloisomerization reaction followed by a proton transfer step [17]. In the presence of the iridium-π-allyl-amino-dipole intermediate **8**, bisbenzannulated spiroaminals **5** are obtained in an extremely stereoselective manner (8:1–12:1 dr, >98% ee). Unlike the previous example, this challenging Ir/Ag/acid ternary catalysis does not lead to kinetic resolution of the starting 2-(1-hydroxylallyl)anilines **7**. Moreover, mechanistic investigations and theoretical calculations revealed that the enantioselectivity (allylation step) was controlled by the chiral iridium catalyst, while the diastereoselectivity was attributed to thermodynamically promoted spiroamination, catalyzed by a Brønsted acid.

**Scheme 8:**
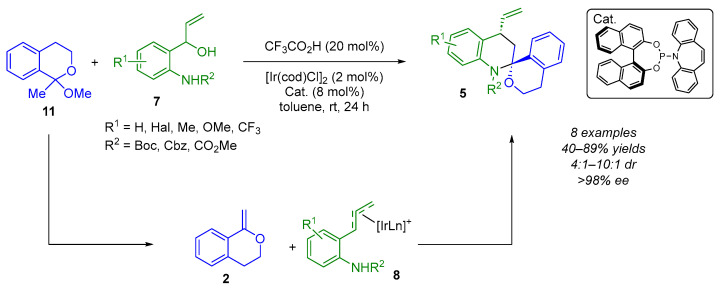
Enantioselective synthesis of spiroaminals from 2-(1-hydroxylallyl)anilines and *iso*chromane acetals.

Despite the flexibility and modularity of the precedented reported methodologies, the use of a bi- or trimetallic catalytic system increases the complexity of the process. Indeed, all of the above examples use exocyclic enol ethers or exocyclic enamines **2** formed in situ via metal catalysis. However, in the following example, Yang and Zheng used isochromane ketals **11**, which produce exocyclic enol ethers **2** in an acidic environment, eliminating the need for a metal-catalyzed cycloisomerization reaction (Scheme 8) [18]. In the presence of [Ir(cod)Cl]_2_, Carreira’s P-olefin ligand and CF_3_CO_2_H, the cascade reaction proceeded smoothly, providing the (*N*,*O*)-spiroketal products **5** with moderate diastereoselectivities (4:1–10:1 dr) and excellent enantioselectivities (>98% ee) in 40–89% yields.

**Scheme 9:**
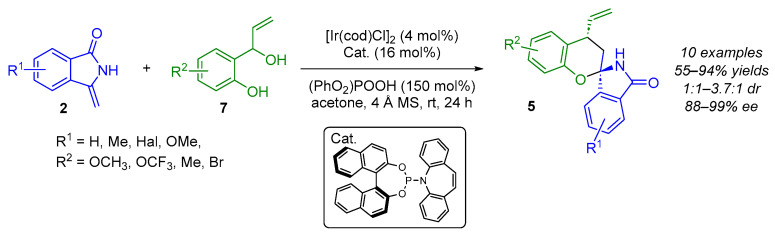
Enantioselective synthesis of spiroaminals from 2-(1-hydroxylallyl)phenols and 3-methylene isoindolones.

In 2024, Liu and Li developed a similar strategy involving 3-methylene isoindolones **2** (Scheme 9) [19]. These compounds are interesting because they are easily accessible and stable enamide moieties. After determining the optimal conditions, the authors demonstrated that this strategy could efficiently produce the desired spiroaminal moieties **5**, regardless of the nature of the substituents (electron-withdrawing, electron-donating or bulky groups) present on the isoindolinones **2** and/or pre-amphiphilic 2-(1-hydroxyallyl)phenols **7**.

### 2.3. Metallocatalyzed (3 + 2) Annulation Reaction

**Scheme 10:**
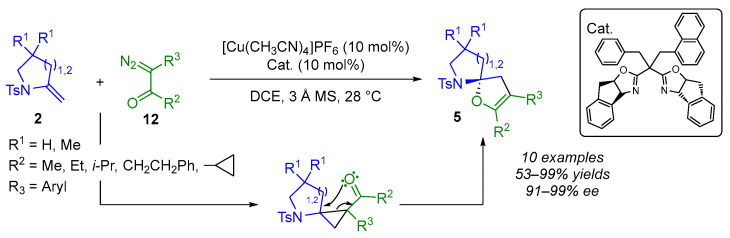
Copper-catalyzed enantioselective CP-RA reaction involving exocyclic enamides and α-aryl-α-diazoketones.

Another proven strategy for accessing enantiomerically enriched (*N*,*O*)-spiroketal moieties is based on a process consisting of a cyclopropanation reaction followed by a rearrangement (CP-RA). Tang and Wang developed a copper-catalyzed enantioselective CP-RA reaction involving exocyclic enamides **2** and α-aryl-α-diazoketones **12**, achieving high levels of selectivity (Scheme 10) [20]. Indeed, both α-diazoketones **12**, bearing various carbonyl substituents (such as acetyl, propionyl and isopropyl groups), and five- or six-membered exocyclic enamides **2** have been shown to be suitable substrates. The corresponding products **5** were obtained in good yields (53–99%) and with high enantiomeric excesses (91–96% ee).

**Scheme 11:**
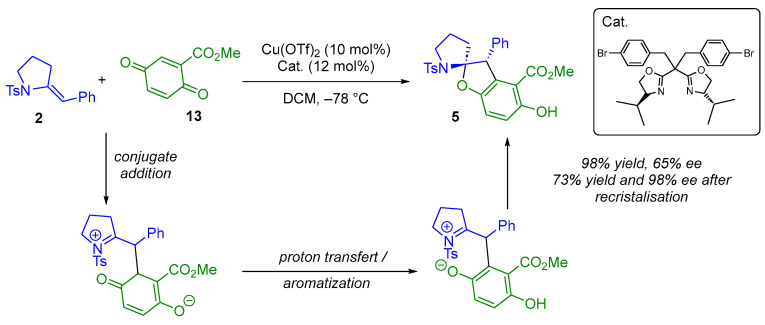
Copper-catalyzed enantioselective (3 + 2) annulation reactions involving exocyclic enamides and 1,4-benzoquinone derivatives.

1,4-Benzoquinone derivatives are readily available starting materials that have been shown to undergo a conjugate addition reaction with nucleophiles. Following proton transfer and aromatization steps, the resulting phenolate anions formed in situ can engage in a cyclization reaction to produce oxygen-containing five-membered rings. In this context, the same group developed a copper-catalyzed series of annulation reactions using 1,4-benzoquinone derivatives (Scheme 11) [21,22,23]. More specifically, the authors studied the racemic reaction of five- or six-membered exocyclic enamides and sulfonylenamides **2** with 1,4-benzoquinone derivatives **13** in order to construct (*N*,*O*)-spiroketal moieties **5** via (3 + 2) annulation reactions. The authors validated their methodology by reporting an enantioselective example. Using a chiral bisoxazoline ligand, they obtained the desired product with a yield of 98% and an enantiomeric excess of 65% (98% after recrystallization) [23].

### 2.4. Lewis Acid Catalysis

**Scheme 12:**
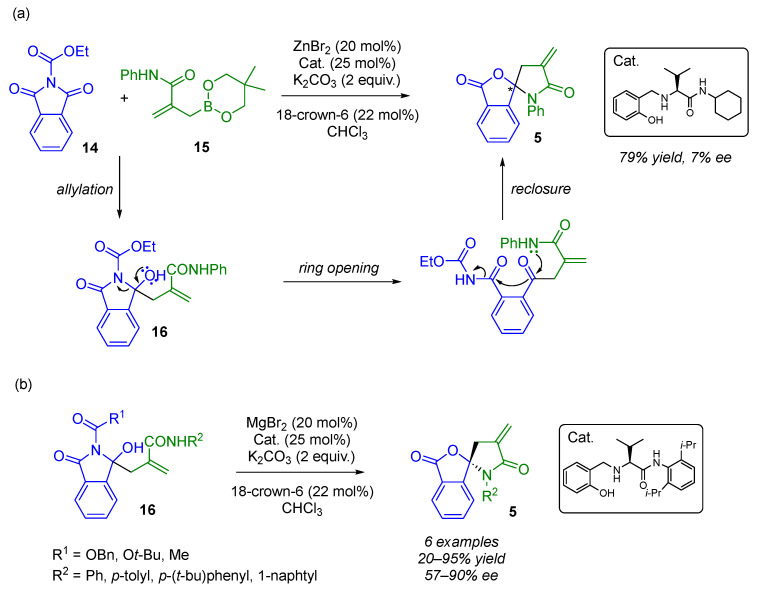
(**a**) Lewis acid-catalyzed reaction cascade involving γ-hydroxylactam derivatives and allylboronates. (**b**) Ring opening–enantioselective reclosure of γ-hydroxylactam derivatives with a methacrylamide side chain.

Yoda described the synthesis of new types of biologically active (*N*,*O*)-spirocyclic compounds. These were formed via a Lewis acid-catalyzed reaction cascade involving the nucleophilic addition of amide-functionalized allylboronates **15** to *N*-carbonylimides **14**, followed by the opening reaction and the closing reaction of the intermediate hydroxylactam ring **16** [24,25,26]. In 2020, the authors attempted to convert the racemic version into an enantioselective one by adding a chiral ligand, but this proved unsuccessful (Scheme 12a) [27]. Therefore, they simplified the system and began working with γ-hydroxylactam derivatives with a methacrylamide side chain **16** (Scheme 12b). Using a new MgBr_2_-chiral aminophenol-based catalyst, Yoda reported an enantioselective ring-opening and -reclosing cascade smoothly enabling the synthesis of enantiomerically enriched (*N*,*O*)-spirocyclic compounds **5** (20–95% yield, 57–90% ee). It was observed that the enantiomeric excess was at its lowest (57% ee) when a bulky substituent, such as *tert*-butyl group, was attached to the nitrogen atom. This would directly impact the coordination of the substrate with the chiral catalyst.

### 2.5. Organocatalysis

**Scheme 13:**
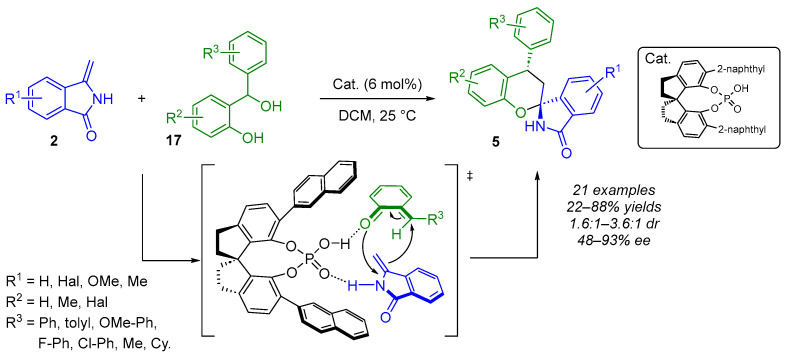
CPA-catalyzed enantioselective synthesis of spiroaminals from *ortho*-hydroxybenzyl alcohols and 3-methylene isoindolones.

*ortho*-Quinone methides (*o*-QMs) typically act as Michael acceptors and serve as versatile synthetic transient intermediates in catalytic asymmetric conjugate addition and (4 + 2) cycloaddition reactions. One way to obtain *o*-QMs is to treat *ortho*-hydroxybenzyl alcohols **17** with a Brønsted acid. From this observation, in 2022, Wang’s group used chiral phosphoric acids as bifunctional catalysts to (1) generate *o*-QMs from *ortho*-hydroxybenzyl alcohols **17** and (2) synthesize enantioenriched (*N*,*O*)-spiroaminal moieties **5** via a formal (4 + 2) cycloaddition reaction in the presence of 3-methylene isoindolinone **2** (Scheme 13) [28]. Indeed, in the optimal conditions, they obtained the desired spiro chroman-isoindolinones containing (*N*,*O*)-spiroketal moieties with moderate diastereoselectivity (1.6:1–3.6:1 dr) and with good enantioselectivities (48–93% ee). During the optimization steps, the authors found that adding the 3-methylene isoindolinone in two batches (60% then 40%) improved the yield of product **5** while maintaining the stereoselectivity. Additionally, the lowest diastereoselectivity (1.6:1 dr) was observed when R^2^ = 4-Br, and the lowest enantioselectivity (48% ee) when R^3^ = *m*-F-Ph. From a mechanistic point of view, the possibility of a concerted mechanism between 3-methylene isoindolinone and *o*-QMs could not be ruled out. However, the authors proposed a transition state assembly for the CPA-catalyzed asymmetric formal (4 + 2) cycloaddition, as they suggested that a stepwise Michael addition and subsequent nucleophilic oxygen addition seem more reasonable.

**Scheme 14:**
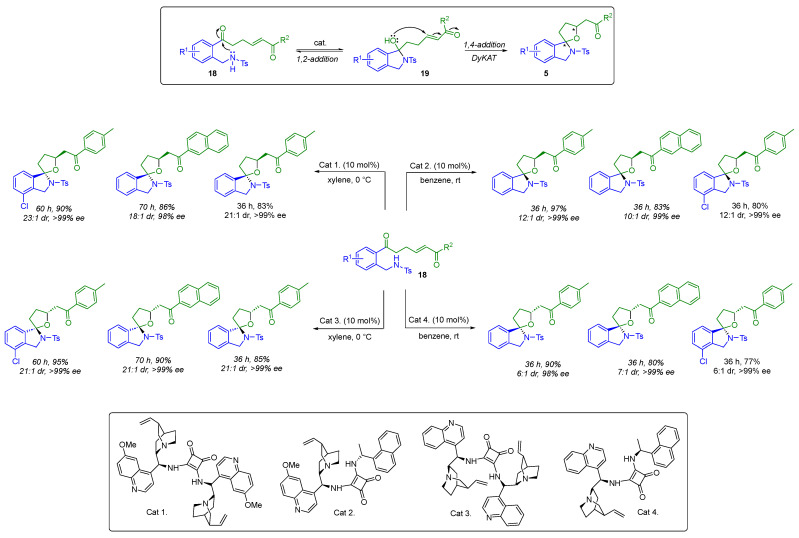
Stereodivergent synthesis of (*N*,*O*)-spiroketals.

In 2024, Ghorai’s group also exploited a non-covalent interaction to achieve enantioselective synthesis of (*N*,*O*)-spiroketal moieties (Scheme 14). Starting with a multifunctional substrate **18**, containing a keto-tethered Michael acceptor unit and a protected amine group, they carried out the first stereodivergent synthesis of these spiroheterocycles by using chiral bifunctional organic catalysts [29]. This stereodivergent strategy is based on reversible intramolecular 1,2-addition of the *N*-protected amine to the carbonyl function, forming the chiral hemiaminal intermediate **19**. This is then followed by an oxa-Michael addition via a dynamic kinetic asymmetric transformation (DyKAT). This cascade spirocyclization successfully produced all four highly enantioenriched stereoisomers of spiroisoindolines from the same starting material by modulating the chiral components of squaramide catalysts with a suitable combination of chiral amines. The authors have demonstrated the power and efficiency of this strategy with more than 60 examples. For greater clarity, Scheme 14 only describes examples leading to all stereoisomers. To prove that the second step of spirocyclization is a DyKAT, the authors synthesized a racemic hemiaminal. Treatment with the optimized catalysts provided all the respective stereoisomers with excellent stereocontrol.

## 3. (*N*,*N*)-Spiroketals

**Figure 2:**
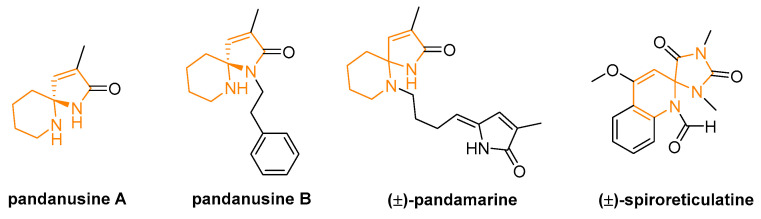
Selected natural products with (*N*,*N*)-spiroketal skeleton.

Compared with (*N*,*O*)-spiroketal units, (*N*,*N*)-spiroketal backbones are less prevalent in natural products (see some examples in Figure 2) and have therefore attracted less interest. Nevertheless, several groups have developed enantioselective methods for these spiroaminal structures with the aim of incorporating them into indole and/or isoindoline frameworks. These frameworks represent indispensable structural motifs in numerous pharmaceutical agents that exhibit a wide range of biological activities.

### 3.1. CPA-Catalyzed (3 + 2) Annulation Reaction

**Scheme 15:**
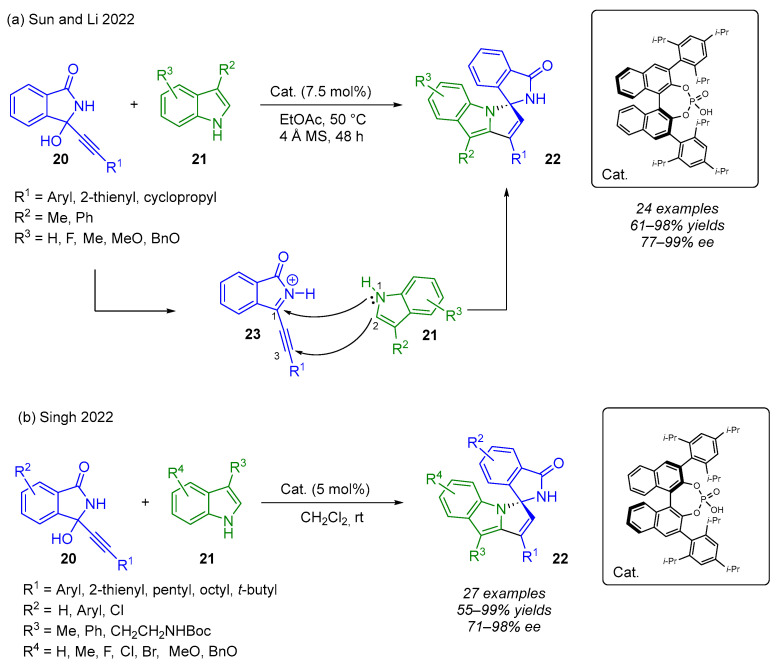
(**a**) Sun and Li organocatalytic (3 + 2) annulation of 3-alkynyl-3-hydroxyisoindolinones with 1*H*-indoles [33]; (**b**) Singh organocatalytic (3 + 2) annulation of 3-alkynyl-3-hydroxyisoindolinones with 1*H*-indoles [34].

Propargylic alcohols are readily available and versatile synthons in organic synthesis. Their unique bifunctional character makes them valuable intermediates in a range of transformations, including substitutions, conjugate additions and annulations, for the efficient construction of diverse, functionalized molecular architectures [30,31]. Recent advances in stereoselective organocatalysis have enabled the enantioselective transformation of propargylic alcohols, particularly α-functionalized propargylic alcohols (α-FPAs) [32]. More precisely, chiral phosphoric acids (CPAs) and their analogs facilitate the in situ dehydration of α-FPAs to generate electron-deficient intermediates, which undergo highly enantioselective additions. Building on their previous work in CPA-catalyzed reactions of propargylic alcohols, Li and Sun became interested in exploring indolinones as α-auxiliary groups. They achieved an organocatalytic (3 + 2) annulation of 3-alkynyl-3-hydroxyisoindolinones **20** with 1*H*-indoles **21** affording spiro-isoindolinone-indoline moieties **22** (Scheme 15a) [33]. Strategically, the in situ-generated β,γ-alkynyl-α-ketimine intermediate **23** is comparable to 1,3 di-electrophiles and the 1*H*-indoles as 1,2 nucleophiles. Regardless of the nature of the substituent R^1^, R^2^ and R^3^, the (*N*,*N*)-spiroketal compounds were obtained in good yield (61–98%) and with high enantioselectivity (77–99%). The lowest enantioselectivity (77%) was observed with the cyclopropyl alkyne-derived substrate. In a subsequent study, Singh and coworkers described a related transformation (Scheme 15b). Using slightly modified conditions, they observed the same yields and the same enantioselectivities with similar alkyl groups [34].

**Scheme 16:**
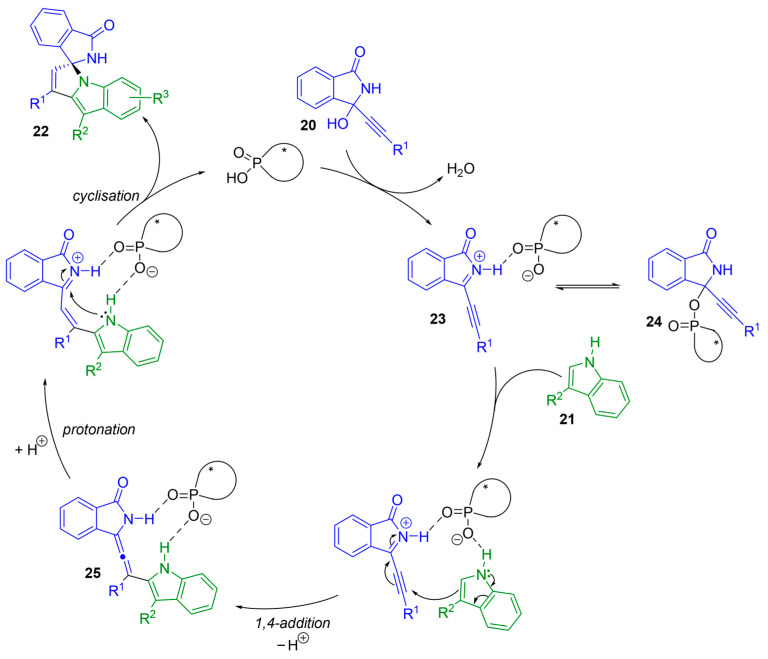
Proposed mechanism.

Based on control experiments and mechanistic studies, the authors proposed the following mechanism based on ion-pair and hydrogen-bonding catalysis (Scheme 16). The first step would be the formation of the acyliminium **23**. In contrast to Singh’s finding, Sun and Li observed the formation of a covalent phosphate ester intermediate **24** by ^1^H NMR, which would act as a pre-catalyst. This would be followed by a 1,4-addition reaction furnishing tetra-substituted allene **25**. Protonation of **25**, followed by intramolecular cyclization, would lead to the desired product **22**. Interestingly, when 3-unsubstituted 1*H*-indole is used, the 1,2-addition takes place instead of the 1,4-addition reaction.

The presence of multiple stereogenic elements within a molecule can have a significant impact on its chemical, biological and physical properties. These fascinating molecules are not only encountered in nature, but also designed for a variety of applications, including use as organocatalysts and ligands in enantioselective catalysis. Consequently, molecules bearing multiple stereogenic elements have attracted significant interest, as their distinct three-dimensional features can be exploited in many different areas [35].

**Scheme 17:**
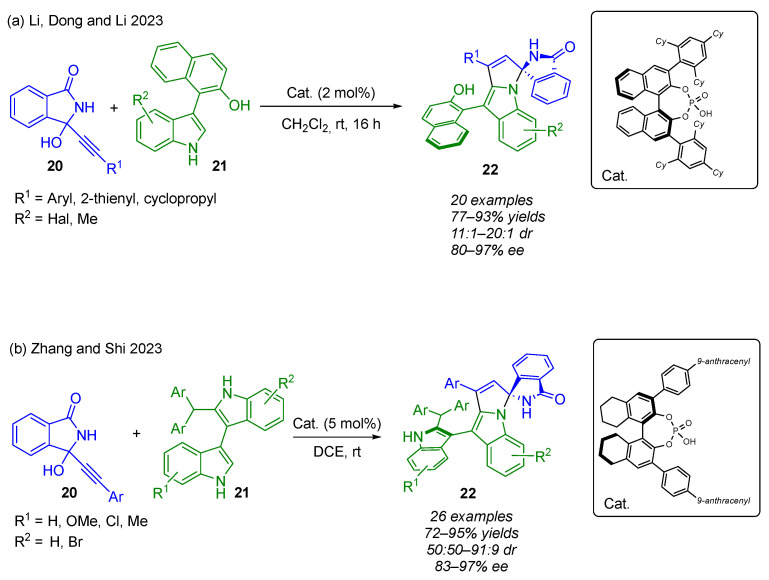
(**a**) Li, Dong and Li (3 + 2) annulation: synergistic construction of axial and central chirality [36]; (**b**) Zhang and shi (3 + 2) annulation: synergistic construction of axial and central chirality [37].

In this context, Li, Dong and Li successfully developed an organocatalytic approach for the construction of both axial and central chirality thanks to a similar CPA-catalyzed (3 + 2) annulation between 3-alkynyl-3-hydroxyisoindolinones (α-FPAs) **20** and 1-(3-indolyl)naphthalen-2-ols **21** (Scheme 17a) [36]. This strategy allowed the smooth construction of enantioenriched (*N*,*N*)-spiroketal moieties **22**, bearing both one stereogenic center and one chirality axis, with excellent diastereo- and enantioselectivities (77–93% yield, 11:1–20:1 dr and 80–97% ee). As previously stated, when R^1^ is a cyclopropyl group, the stereoselectivity is drastically affected, leading to the formation of a racemic product. At the same time, Zhang and Shi reported the same strategy with the same efficiency starting from 3,3-bisindoles **21** as 1,2-dinucleophiles (Scheme 17b) [37].

### 3.2. Photocatalytic Process

**Scheme 18:**
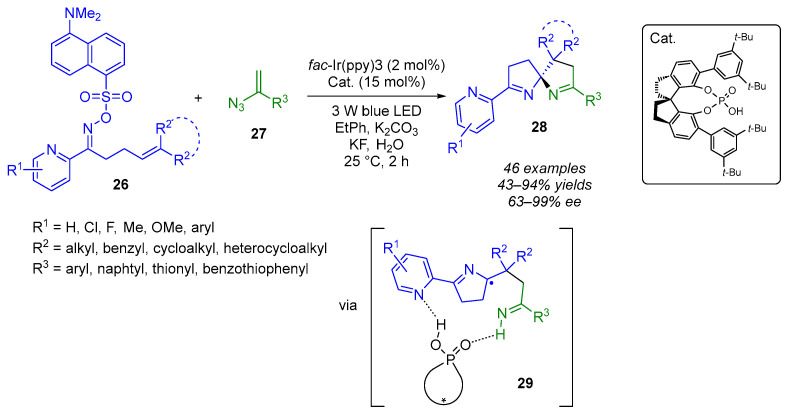
Enantioselective radical addition.

Chiral hydrogen-bond catalysis has been successfully applied to a wide range of stereoselective photocatalytic radical-based reactions. A critical problem for these processes is the formation of secondary anionic intermediates that interact preferentially with the protons of chiral catalysts, thus compromising the enantioselective control. The key innovation brought about by Jiang’s group lies in modulating the redox mechanism (single-electron transfer, SET) towards an energy transfer (EnT) process. This adjustment shifts the generation of anionic intermediates in chiral catalysis by hydrogen bonding to a stage after the formation of stereogenic centers. The result is an enhanced preferential interaction between acid catalysts and key radical species. The researchers have succeeded in carrying out the first stereoselective photochemical spirocyclization reaction for building spiro quaternary stereogenic centers through enantioselective radical addition (Scheme 18) [38]. Through use of a dual-catalysis system that combines a photosensitizer and a chiral Brønsted acid, a wide range of olefinic sulfonyl oximes **26** can be efficiently reacted with vinyl azides **27** to produce azaarene-functionalized (*N*,*N*)-spiroketals **28** in good yields (43–94%) and with excellent enantioselectivity (63–99%). The two hydrogen-bonding interactions between the chiral CPA and azarene **29** would be responsible for the enantiocontrol of the 5-*endo*-trig cyclization step. It is worth noting that the lowest enantioselectivity was observed when R^2^ was a cyclobutyl group. The incorporation of a sulfonyl protecting group on the oximes enables these transformations to be initiated via an EnT process rather than the SET approach used previously [39].

## 4. (*N*,*S*)-Spiroketals

Sulfur-containing heterocyclic frameworks are commonly found in biologically active molecules and are associated with a wide range of pharmacological properties. Among them, benzothiophene and its derivatives have emerged as particularly interesting structural frameworks. Therefore, significant synthetic efforts have been devoted to developing and diversifying the structure of benzothiophene-based compounds. In addition, introducing fluorine into bioactive molecules can result in significant changes to their chemical, physical and biological properties. The fluorine atom uniquely affects the properties of organic molecules due to its blocking effect in metabolic transformations and its ability to mimic enzyme substrates, increasing their lipophilicity and enhancing bioavailability. Consequently, organofluorine compounds have attracted considerable attention in the development of new drugs and agrochemicals.

**Scheme 19:**
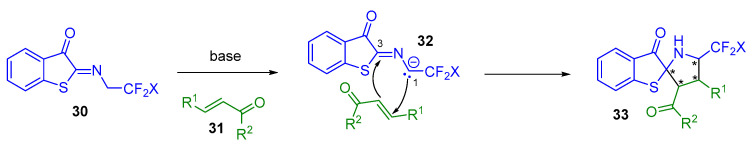
Enantioselective (3 + 2) annulation reactions with trifluoro- or difluoroethyl benzothiophene ketimines.

In this context, several groups have employed trifluoro- or difluoroethyl benzothiophene ketimines **30** in enantioselective (3 + 2) annulation reactions to construct enantioenriched CF_3_- or CF_2_H-embedded spiro-benzothiophenone **33** (Scheme 19). Indeed, in the presence of a base, which, for the purposes of the following examples, is a tertiary amine carried by a bifunctional squaramide or thiourea catalyst, benzothiophene ketimines **30** are transformed into a 1,3-amphiphilic intermediate **32** and can therefore react with a α,β-unsaturated ketone **31** to form (*N*,*S*)-spiroketal units **33** with up to four stereogenic centers. This strategy can be associated with either a two-step mechanism or a concerted 1,3-dipolar (3 + 2) cycloaddition reaction.

**Scheme 20:**
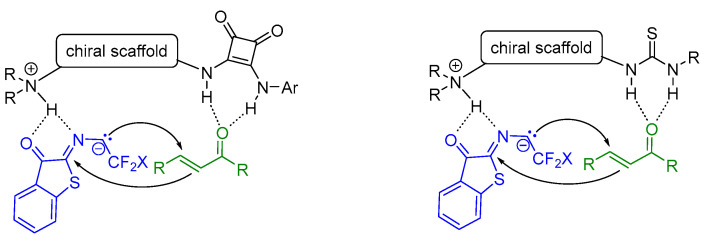
Dual-activation model.

The stereoselectivity of this reaction can be explained by a dual-activation model of the catalyst, whereby both substrates are simultaneously activated via hydrogen bonds (Scheme 20). The carbanion formed by the catalyst deprotonating benzothiophene ketimines coordinates with the protonated cinchonidin nitrogen atom to form a five-membered ring. At the same time, the two squaramide or thioamide N–H bonds of the catalyst coordinate with the α,β-unsaturated ketone. The chiral catalyst brings the two substrates close together, inducing a highly stereoselective reaction that delivers the desired product.

**Scheme 21:**
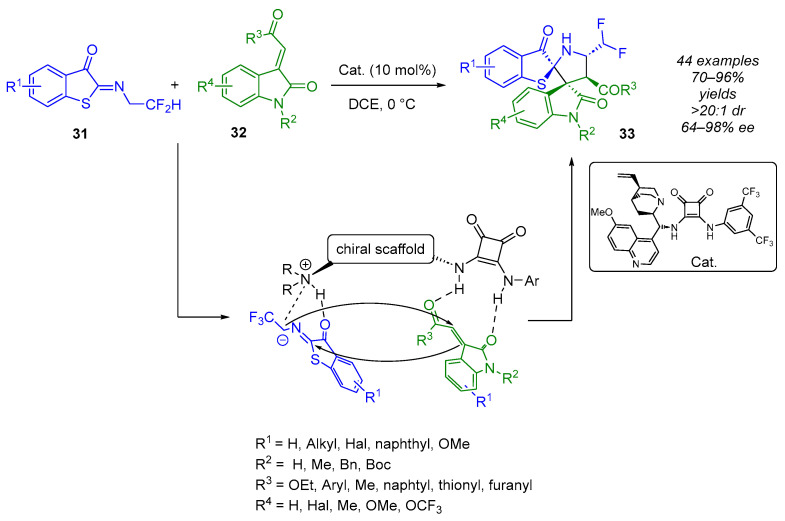
Squaramide-catalyzed enantioselective (3 + 2) cycloaddition reaction between the *N*-(2,2)-difluoroethyl benzothiophene ketimine and the 2-oxoindolin-3-ylidene acetate.

In 2022, the group of Yan described a squaramide-catalyzed enantioselective (3 + 2) cycloaddition reaction between the *N*-(2,2)-difluoroethyl benzothiophene ketimine **30** and the 2-oxoindolin-3-ylidene acetate **31** (Scheme 21) [40]. Under optimized conditions, they obtained the desired CF_2_H-containing bispiro(oxindole-pyrrolidine-benzothiophenone)s **33** with four adjacent stereogenic centers, including two adjacent spirocyclic tetra-substituted stereocenters. These bispiro-compounds were formed in a single diastereomer (>20:1), in high yields (70–96%) and typically with excellent enantioselectivities (64–98% ee), whatever the nature of the substituents borne by both substrates. The lowest enantioselectivity (64% ee) was observed when R^2^ = H. In this case, the 2-oxoindolin-3-ylidene acetate **31** is not very soluble and the reaction has to be performed at room temperature.

**Scheme 22:**
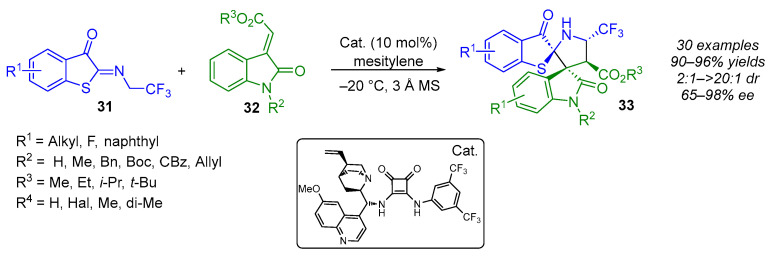
Squaramide-catalyzed enantioselective (3 + 2) cycloaddition reaction between the *N*-(2,2,2)-trifluoroethyl benzothiophene ketimine and the 2-oxoindolin-3-ylidene acetate.

In 2023, in collaboration with Huang, Yan reported a similar squaramide-catalyzed enantioselective (3 + 2) cycloaddition strategy from trifluoroethyl benzothiophene ketimine **30** (Scheme 22) [41]. The CF_3_-containing bispiro(oxindole-pyrrolidine-benzothiophenone)s were obtained with the same efficiency. However, the nature and position of the substituents on both partners significantly affect the diastereoselectivity (2:1–>20:1 dr).

**Scheme 23:**
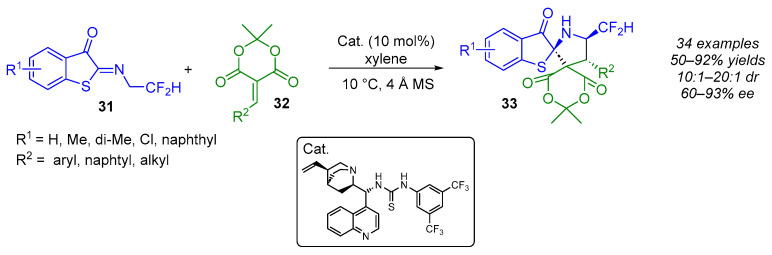
Thiourea-catalyzed enantioselective (3 + 2) cycloaddition reaction between the *N*-(2,2)-difluoroethyl benzothiophene ketimine and the Meldrum’s acid derivatives.

As part of their ongoing interest in the synthesis of optically pure F-containing pyrrolidines, the same group reported in 2023 on the use of Meldrum’s acid-derived electron-deficient olefins as dipolarophiles in enantioselective (3 + 2) cycloaddition reactions (Scheme 23) [42]. In the presence of a cinchonidine-thiourea-based bifunctional catalyst, this approach produced highly enantioenriched (60–93% ee) α-CF_2_H pyrrolidines embedded in (*N*,*S*)-spiroketals with three adjacent stereogenic centers in good yields (50–92%) and excellent diastereoselectivity (10:1–20:1 dr). The lowest enantioselectivity (60%) was obtained when an electron-withdrawing group (R^1^ = Cl) was present on the 1,3-dipole. Additionally, the use of aliphatic chain-substituted Meldrum’s acid affected the diastereoselectivity (10:1 and 12:1 dr) and the yield (50% and 70%) of the reaction.

**Scheme 24:**
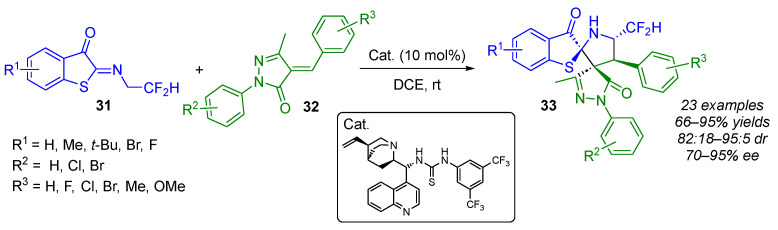
Thiourea-catalyzed enantioselective (3 + 2) cycloaddition reaction between the *N*-(2,2)-difluoroethyl benzothiophene ketimine and pyrazolone derivatives.

Enantiopure spiropyrazolones are well recognized for their diverse and valuable biological activities. Building on this, Li, Huang and Yan have recently applied their expertise in organocatalyzed (3 + 2) cycloaddition reactions of 1,3-dipoles derived from *N*-(2,2-difluoroethyl) benzothiophene ketimines **31** to synthesize optically active dispiro[benzo[b]thiophene-pyrrolidine-pyrazole] derivatives **33** bearing a (*N*,*S*) acetal moiety (Scheme 24) [43]. Indeed, the authors demonstrated that unsaturated pyrazolone derivatives **32** were effective partners in this enantioselective (3 + 2) cycloaddition reaction using a bifunctional thiourea catalyst. Cycloadducts, containing two adjacent spiral units, including one (*N*,*S*)-ketal moiety and four contiguous stereogenic centers, were obtained in good yields (66–95%) with general diastereoselectivities (82:18–95:5 dr) and with good-to-high enantioselectivities (70–95% ee).

Two other groups have also recently studied this organocatalyzed (3 + 2) cycloaddition reaction in order to produce spiroacetal (*N*,*S*) units from benzothiophene ketimines.

**Scheme 25:**
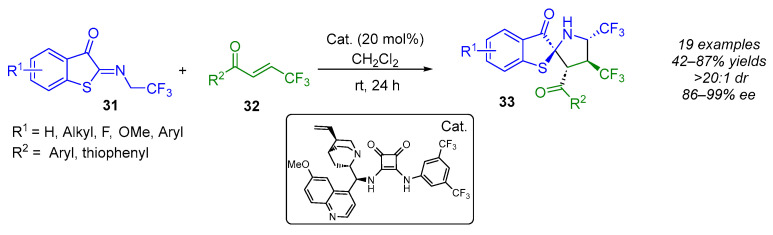
Squaramide-catalyzed enantioselective (3 + 2) cycloaddition reaction between the *N*-(2,2,2)-trifluoroethyl benzothiophene ketimine and β-trifluoromethyl enones.

In 2023, Yuan’s group used β-trifluoromethyl enones **32** as a dipolarophile in an enantioselective (3 + 2) cycloaddition reaction involving trifluoroethyl benzothiophene ketimines **31** (Scheme 25) [44]. This strategy employed a chiral bifunctional squaramide–tertiary amine catalyst to prepare challenging and potentially bioactive vicinally bis(trifluoromethyl)-substituted (*N*,*S*)-spiroketals bearing four contiguous stereogenic centers with excellent stereocontrol (86–99% ee, >20:1 dr).

**Scheme 26:**
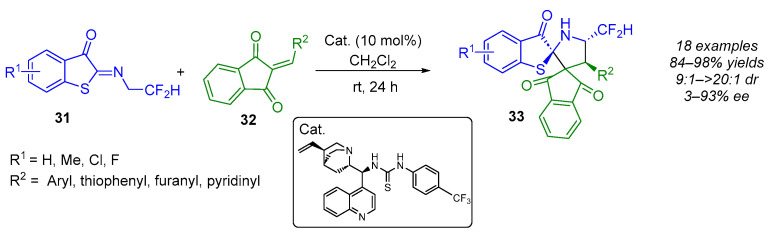
Thiourea-catalyzed enantioselective (3 + 2) cycloaddition reaction between the *N*-(2,2)-difluoroethyl benzothiophene ketimine and 2-arylidene-1,3-diones.

Finally, Du demonstrated the efficiency with which 2-arylidene-1,3-diones [45,46] react with *N*-2,2-difluoroethyl benzothiophenone imines in this type of reaction (Scheme 26) [47]. Under mild conditions, the thiourea-catalyzed enantioselective (3 + 2) cycloaddition produced a range of dispiro[benzothiophenone-indandione-pyrrolidine] products with excellent yields (84–98%). It is worth noting that the stereoselectivity is influenced by the nature of the substituents on both substrates. Indeed, the dispiro-derivatives display enantioselectivities of 3–93% ee and diastereomeric ratios ranging from 9:1 to 20:1.

## 5. Conclusions

In this review, we describe progress in the field of enantioselective synthesis of (*N*,*O*)-, (*N*,*N*)- and (*N*,*S*)-spiroketals. The strategies are mainly based on metallo- and organocatalyzed (4 + 2) and (3 + 2) annulation reactions, Lewis acid-catalyzed cascade reactions and photocatalytic radical-based reactions.

## Data Availability

Not applicable.

## Abbreviations

The following abbreviations are used in this manuscript:

IED hetero-DA reaction	Inverse Electron-Demand Hetero-Diels–Alder Reaction
CP-RA	Cyclopropanation Reaction–Rearrangement
DyKAT	Dynamic Kinetic Asymmetric Transformation
CPA	Chiral Phosphoric Acid
*ortho*-Quinone Methides	*o*-QMs
α-Functionalized Propargylic Alcohols	α-FPAs
SET	Single-Electron Transfer
EnT	Energy Transfer
ee	Enantiomeric Excess
dr	Diastereomeric Excess
rt	Room Temperature
MS	Molecular Sieves
cat	Catalyst
